# A Cu^2+^-doped two-dimensional material-based heterojunction photoelectrode: application for highly sensitive photoelectrochemical detection of hydrogen sulfide

**DOI:** 10.1039/c9ra05385a

**Published:** 2019-09-09

**Authors:** Siyuan Yu, Xia Chen, Chaobiao Huang, Deman Han

**Affiliations:** College of Chemistry and Life Sciences, Zhejiang Normal University Jinhua 321004 China hcb@zjnu.cn; Department of Chemistry, Taizhou University Jiaojiang, 318000 China hdmtzc@126.com

## Abstract

In this work, on the basis of a Cu^2+^-doped two-dimensional material-based heterojunction photoelectrode, a novel anodic photoelectrochemical (PEC) sensing platform was constructed for highly sensitive detection of endogenous H_2_S. Briefly, with g-C_3_N_4_ and TiO_2_ as representative materials, the sensor was fabricated by modifying g-C_3_N_4_/TiO_2_ nanorod arrays (NAs) onto the surface of fluorine-doped tin oxide (FTO) and then doping Cu^2+^ as a Cu_*x*_S (*x* = 1, 2) precursor. After the binding of S^2−^ with surface-attached Cu^2+^, the signal was quenched owing to the *in situ* generation of Cu_*x*_S which offers trapping sites to hinder generation of photocurrent signals. Since the photocurrent inhibition was intimately associated with the concentration of S^2−^, a highly sensitive PEC biosensor was fabricated for H_2_S detection. More importantly, the proposed sensing platform showed the enormous potential of g-C_3_N_4_/TiO_2_ NAs for further development of PEC bioanalysis, which may serve as a common basis for other semiconductor applications and stimulates the exploration of numerous high-performance nanocomposites.

## Abstract 2

In this work, on the basis of a Cu^2+^-doped two-dimensional material-based heterojunction photoelectrode, a novel anodic photoelectrochemical (PEC) sensing platform was constructed for highly sensitive detection of endogenous H_2_S.

## Graphical abstract

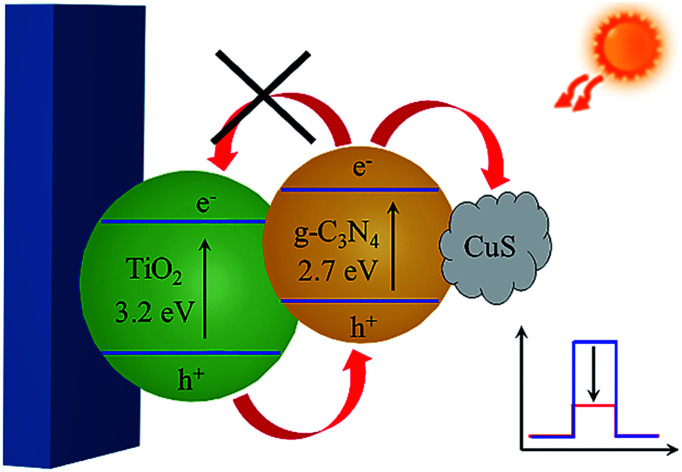

## 1. Introduction

Since hydrogen sulfide (H_2_S) was found to be the third endogenously generated gaseous signaling molecule following nitric oxide and carbon monoxide with cytoprotective properties, great attention has been drawn in the field of clinical diagnostics.^1–4^ In addition, H_2_S has also been known to play a crucial role in a series of physiological processes, including antioxidation,^5^ anti-inflammation^6^ and apoptosis.^7^ On the other hand, when the release concentration of H_2_S in the atmosphere is greater than the olfactory perception threshold of 300 ppb, it will harm human health and induce nausea, headaches, and lung irritation.^8^ Even chronic, low-level exposures can also lead to irreversible health effects.^9,10^ From this point of view, there is an essential demand to develop a reliable and high-performance approach for H_2_S monitoring.

Photoelectrochemical bioanalysis represents an elegant route for highly sensitive detection and exhibits versatile advantages of decreased costs, simple sample preparation, high sensitivity and selectivity,^11–18^ which has inspired the rapid development of this field in recent years. Previously, many PEC analytical methods have been exploited for H_2_S detection.^19–24^ The most common strategy is *in situ* generated CdS on the surface of TiO_2_ to enhance the photocurrent response.^20–24^ But the strategy of *in situ* sensitization *via* CdS has its own limitations on only fitting for the PEC substrates with low photoelectrical activity. And to the best of our knowledge, few works had been conducted for H_2_S detection with the strategy of *in situ* quenching.

2D materials have been among the most important research hotspots in the past years for their superlative physical properties and manifold implications in various fields. These materials consist of atomically thin sheets with large specific surface area exhibiting covalent in-plane bonding and weak interlayer and layer–substrate bonding. Besides, 2D materials can not only display improved inherent properties of the bulk materials but also give birth to new properties that the corresponding bulk materials do not possess.^25–27^ On the other hand, in pursuit of achieving better semiconducting performances, heterostructures comprised by different semiconductors are being considered as favorite schemes as compared to the pure ones. It is believed that such a structure could integrate different properties of the individual semiconductors and thus generate enhanced properties.^28–32^ Hence, of particular interest here is the possibility of utilizing ingenious 2D material-based heterojunction for innovative PEC detection of S^2−^. We hypothesize that such a PEC platform possesses great potential in improving performance of PEC detection of S^2−^. If possible, the great enhancement of light-harvesting efficiency is benefiting from the feature of 2D material with large surface area, meanwhile, heterojunction is taken fully advantages of the contribution to the photoinduced charge separation in both the semiconductors and inhibition of the charge recombination, resulting in the improvement in photocurrent generation.^33,34^

**Scheme 1:**
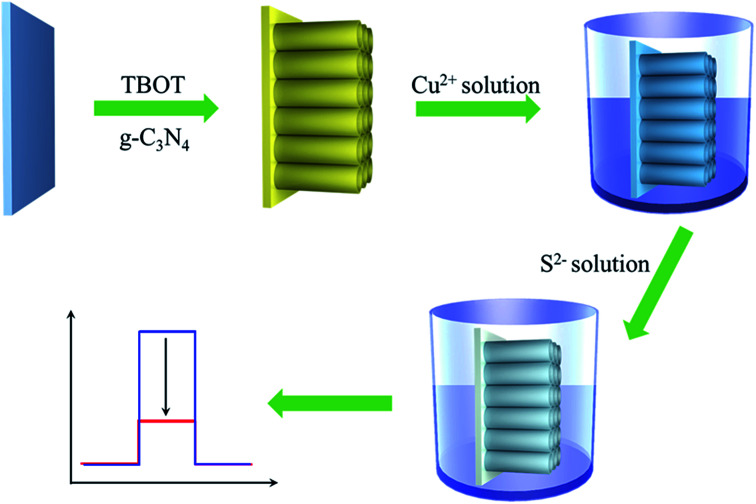
Schematic illustration for the proposed PEC sensing platform.

To verify this hypothesis, with g-C_3_N_4_ and TiO_2_ as representative materials, herein, we put forward a novel and general PEC sensing platform for highly sensitive detection of H_2_S through modifying FTO substrate with Cu^2+^-doped g-C_3_N_4_/TiO_2_ NAs (Scheme 1). In this work, different from the previous strategies for PEC sensors of H_2_S, the obvious photocurrent quenching is appeared upon exposure to S^2−^, owing to *in situ* formed Cu_*x*_S (*x* = 1, 2) has a much lower conduction band edge than g-C_3_N_4_ and offers plentiful surface recombination centers.^35,36^ Thereby, in the presence of S^2−^, the photocurrent intensity is expected to have an evident slip and by monitoring the reduction of photocurrent, we could quantitatively determine the concentration of S^2−^.

## 2. Experimental

### 2.1 Materials and reagents

Fluorine-doped tin oxide (FTO) glass substrate with a thickness of 1.1 mm (sheet resistance ≤ 15 Ω per square) was ordered from South China Science & Technology Co. Ltd. Urea (CO(NH_2_)_2_), tetrabutyl titanate (C_16_H_36_O_4_Ti), hydrochloric acid (HCl), acetone (C_3_H_6_O), anhydrous ethanol (C_2_H_5_OH), copper sulfate (CuSO_4_) and triethanolamine (TEOA) were all purchased from Sinopharm Chemical Reagent Co. Ltd. All other reagents were of analytical grade and used as received. Additionally, all aqueous solutions were prepared with deionized water (DI water, 18 MΩ cm^−1^), which was obtained from a MilliQ water purification system.

### 2.2 Synthesis of g-C_3_N_4_ nanosheets

10.0 g of urea was put into an alumina crucible with a cover, heated with a ramp rate of 10 °C min to 550 °C in air atmosphere in a muffle furnace, and maintained for 4 h. Afterward, the resulting pale yellow agglomerate was milled into powder by a mortar and the g-C_3_N_4_ nanosheets were obtained by liquid exfoliation of the bulk g-C_3_N_4_ powder in water. Briefly, the bulk g-C_3_N_4_ powder was dispersed into 100 mL of distilled water and ultrasonicated for 2 h. The residual unexfoliated bulk g-C_3_N_4_ was removed by centrifugation at 4500*g*. Subsequently, the supernatant was further centrifuged at 8000*g*, and the obtained precipitation was dried at 70 °C in a oven.

### 2.3 Preparation of the biosensor

The g-C_3_N_4_/TiO_2_/FTO was first prepared as follows using a one-step hydrothermal method. First, 10 mg of as-prepared g-C_3_N_4_ powder was homogeneously dispersed in 6 mL of ultrapure water *via* sonication, and the obtained suspension was mixed with equal volume of concentrated hydrochloric acid. After 5 min stirring, 200 μL of tetrabutyl titanate (TBOT) was added into the above suspension and stirred for 30 min. Then the above mixture was transferred into a Teflon-lined stainless steel autoclave. Subsequently, pieces of cleaned FTO substrate were placed against the wall of the autoclave with conductive sides facing down. The autoclave was kept in an oven at 150 °C for 10 h and then allowed to cool down to room temperature. Finally, the FTO substrates were removed, rinsed with ultrapure water, put into a muffle furnace and annealed at 450 °C for 1 h to form TiO_2_/g-C_3_N_4_ NAs on the FTO substrate surface. To prepare Cu_*x*_S precursor, the obtained g-C_3_N_4_/TiO_2_/FTO was immersed into 1.0 mM CuSO_4_ solution with 1 h gently shaking to dope Cu^2+^ on electrode surface.

### 2.4 PEC measurement

The as-prepared PEC sensing platform was exposed to different concentration of Na_2_S solution (H_2_S in the aqueous medium) for 10 min. Followed by washing with ultrapure water thoroughly to remove excess S^2−^. After that, the resulted substrate was transferred into the PEC detection cell for photocurrent measurement.

## 3. Results and discussion

### 3.1 Materials characterization

**Fig. 1:**
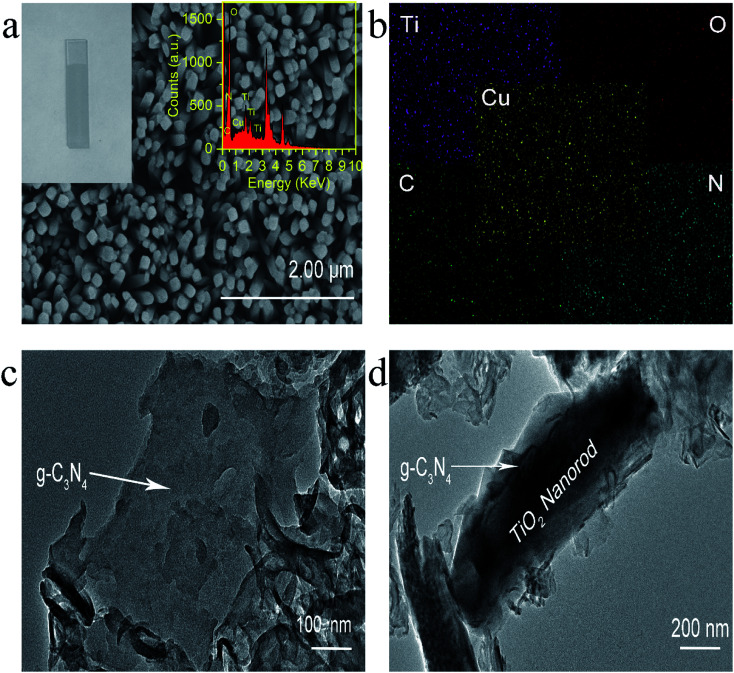
(a) Typical top-view SEM images of Cu^2+^-doped g-C_3_N_4_/TiO_2_ NAs; (b) elemental mapping of Ti, O, C, N and Cu; TEM images of (c) pure g-C_3_N_4_ and (d) g-C_3_N_4_/TiO_2_ heterojunction. Inset in (a): (left) visual photograph of the as-prepared g-C_3_N_4_/TiO_2_/FTO, (right) EDX spectrum of Cu^2+^-doped g-C_3_N_4_/TiO_2_ NAs.

Experimentally, g-C_3_N_4_ and g-C_3_N_4_/TiO_2_/FTO were prepared through a thermo-polymerization method^32^ and a modified hydrothermal method according to a previous report.^37^ The structural and morphology information of the as-prepared g-C_3_N_4_/TiO_2_ NAs were characterized by scanning electron microscopy (SEM), transmission electron microscopy (TEM). As exhibited in Fig. 1a, Cu^2+^-doped g-C_3_N_4_/TiO_2_ NAs were vertically grown on the surface of FTO and g-C_3_N_4_ could not distinctly tell *via* SEM. As indicated in the left inset of Fig. 1a, white g-C_3_N_4_/TiO_2_ film shows the great affinity to FTO. As shown in the right inset of Fig. 1a, the energy dispersive X-ray spectrum (EDX) of Cu^2+^-doped g-C_3_N_4_/TiO_2_/FTO distinctly verify the existence of Ti, O, C, N and Cu elements. The strong peaks for O and Ti elements were found in the spectrum, due to its abundant amount in the composites. Moreover, the presence of Cu in the spectrum revealed that the doping was successful. Fig. 1b of elemental mapping also indicates a uniform distribution of Ti, O, C, N and Cu elements in the sample. Through the TEM image, a typical planar sheet-like the exfoliated g-C_3_N_4_ sample with an irregular shape was displayed in Fig. 1c. In addition, Fig. 1d indicates TiO_2_ NRs was fully wrapped by g-C_3_N_4_ and the existence of a very close and distinguishable g-C_3_N_4_/TiO_2_ interface, revealing the moderate interfacial contact and successful synthesis of g-C_3_N_4_/TiO_2_ nanohybrid.

**Fig. 2:**
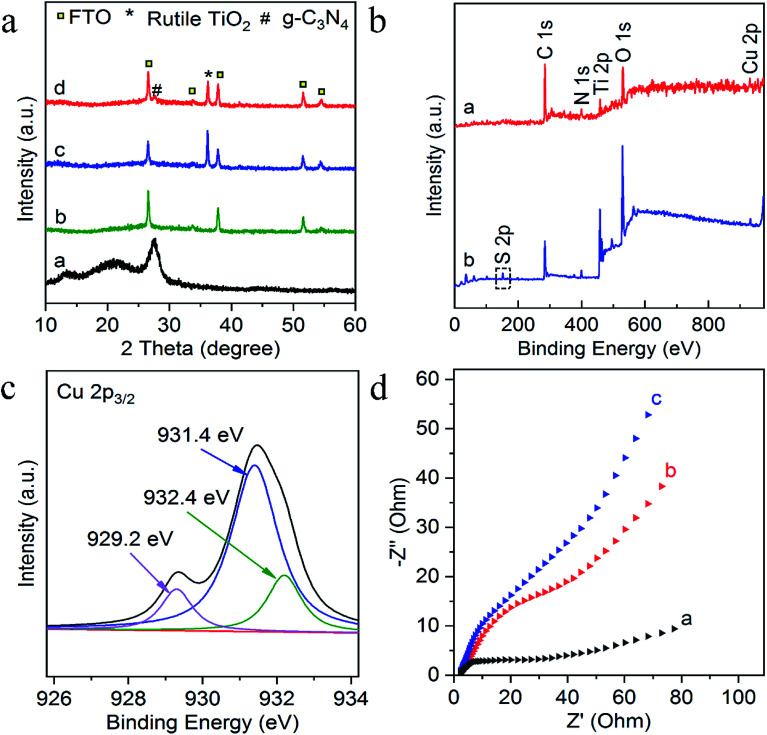
(a) XRD pattern of g-C_3_N_4_ (curve a), bare FTO (curve b), TiO_2_/FTO (curve c) and g-C_3_N_4_/TiO_2_/FTO (curve d); (b) XPS survey spectra of g-C_3_N_4_/TiO_2_ NAs (curve a) and after incubation of S^2−^ (curve b); (c) high resolution XPS spectrum of Cu 2p; (d) EIS spectra of FTO (curve a), g-C_3_N_4_/TiO_2_/FTO (curve b), Cu^2+^-doped g-C_3_N_4_/TiO_2_/FTO after treated with 50 nM S^2−^ (curve c).

The composition and crystal-phase properties of g-C_3_N_4_/TiO_2_/FTO was identified by X-ray diffraction (XRD). As Fig. 2a displayed, two diffraction peaks (curve a) of pristine g-C_3_N_4_ sample at 27.5° and 13.1° corresponds to the (002) and (100) crystal planes, respectively, of graphite-like hexagonal g-C_3_N_4_ (JCPDS no. 87-1526). Diffraction peaks of the bare FTO are clearly shown in curve b. Additionally, the feature peaks of TiO_2_ NAs on FTO at 36.1° (curve c) can be exactly indexed to (101) crystal planes of the well-crystallized rutile phase (JCPDS no. 21-1276). No peaks corresponding to the (110), (111), and (211) crystal planes of the rutile phase were not observed, indicating the growth of TiO_2_ on FTO substrates with high orientation selectivity. No other impurity peaks were detected. As for the g-C_3_N_4_/TiO_2_/FTO (curve d), the feature diffraction at 27.5° could properly match the (002) plane of g-C_3_N_4_ (curve a), implying that g-C_3_N_4_ was successfully modified on the surface of TiO_2_ nanorods. Then as illustrated in Fig. 2b, X-ray photoelectron spectroscopy (XPS) was applied to study the surface chemical compositions and oxidation states of Cu^2+^-doped g-C_3_N_4_/TiO_2_/FTO before (curve a) and after (curve b) reaction with S^2−^. Curve a verifies the presence of C, N, Ti, O and Cu peaks and a new characteristic peak of S 2p emerged, suggesting the presence of sulfur element on the electrode. Fig. 2c reveals the high-resolution XPS spectrum of Cu 2p_3/2_, which could be further distributed into three parts located at 932.4, 931.4, and 929.2 eV. As reported in the previous work,^38^ the main peak at 931.4 eV of Cu^+^ was produced from the interaction between superficial Cu^2+^ and S^2−^, indicating the presence of Cu_*x*_S (*x* = 1, 2) on the surface of g-C_3_N_4_/TiO_2_ NAs. Besides, the one weak peak at 932.4 eV was attributed to CuS,^39^ while the other at 929.2 eV was assigned to the tiny amount of CuO. Combined with above results, Cu_*x*_S was newly formed on the electrode surface after incubation of S^2−^. In addition, the electrochemical impedance spectroscopy (EIS) was also employed to analyze the interfacial properties of the modified electrode in 5 mM [Fe(CN)_6_]^4−/3−^ (as the redox probe) containing 0.1 M KCl. The impedance spectrum includes a semicircular portion at higher frequencies and a linear portion at lower frequencies. The diameter of the semicircle is equal to the electron transfer resistance (*R*_et_). As shown in Fig. 2d, the *R*_et_ value of FTO (curve a) was very small, after coating g-C_3_N_4_/TiO_2_ on electrode surface, *R*_et_ value increased significantly (curve b), which may be due to the introduction of g-C_3_N_4_/TiO_2_ hindered the electron transfer on the electrode. Then, the *R*_et_ value increased after the modified electrode treated with 50 nM S^2−^ (curve c). The reason for the increase in the resistance value were owing to the generation of Cu_*x*_S which offered the trapping sites with new energy levels and thus suppressed the electron transfer on the surface of the g-C_3_N_4_/TiO_2_/FTO.

### 3.2 Optimization of experimental conditions

**Fig. 3:**
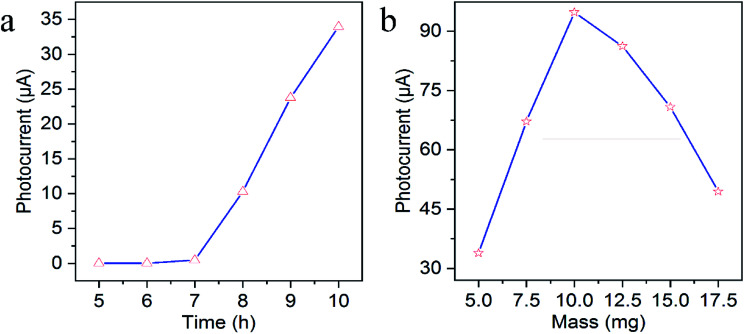
Effects of (a) the hydrothermal reaction time of TiO_2_ NAs and (b) the mass of g-C_3_N_4_ on photocurrent response of g-C_3_N_4_/TiO_2_/FTO.

As PEC sensing performance was affected by some factors influencing the photocurrent response like the length of TiO_2_ NAs and the mass of g-C_3_N_4_, the optimal preparation conditions were conducted. The length of TiO_2_ NAs could be controlled *via* hydrothermal reaction time. As revealed in Fig. 3a, the photocurrent intensity was increasing with the increased reaction time of TiO_2_ NAs. However, if reaction time is extended to over 10 h, the white film composed of aligned TiO_2_ NAs starts to peel off from the FTO substrate during annealing because the length of TiO_2_ NAs was too long to adhere to the FTO substrate consistently. As a result, 10 h is the optimal reaction time associated with maximal photocurrent intensity of TiO_2_ NAs. Fig. 3b shows the effects on photocurrent response of g-C_3_N_4_/TiO_2_/FTO prepared with different mass of g-C_3_N_4_. It can be told that g-C_3_N_4_/TiO_2_/FTO produced the highest photocurrent with 10.0 mg g-C_3_N_4_ added. As the mass grew, g-C_3_N_4_ immobilized on TiO_2_ film gradually increased, thereby leading to wider light absorption range. With more g-C_3_N_4_ added, excessive g-C_3_N_4_ immobilization occurred on TiO_2_ film, which offered more surface recombination centers and the resistance to decrease the photocurrent intensity. Thereby, 10.0 mg of g-C_3_N_4_ was added into the mixture in subsequent experiments.

### 3.3 Characterization of the PEC biosensor

**Fig. 4:**
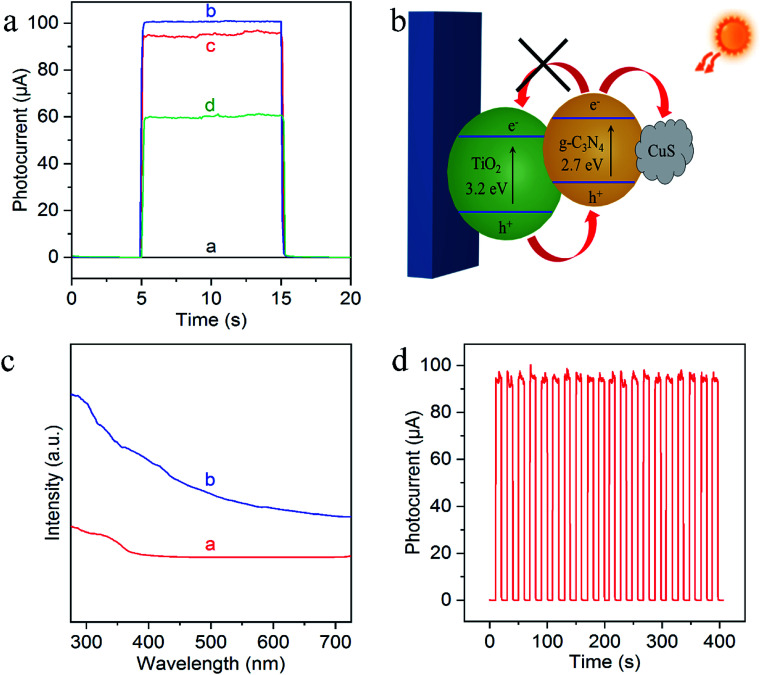
(a) Photocurrent responses of the bare FTO (curve a), g-C_3_N_4_/TiO_2_/FTO (curve b), Cu^2+^-doped g-C_3_N_4_/TiO_2_/FTO (curve c) and after treated with 50 nM S^2−^ (curve d); (b) the proposed mechanism for the decrement of photocurrent response; (c) UV-vis diffuse reflectance spectra of TiO_2_/FTO (curve a) and Cu^2+^-doped g-C_3_N_4_/TiO_2_/FTO (curve b). (d) The operational stability test of g-C_3_N_4_/TiO_2_/FTO by repeated on/off illumination cycles. The PEC tests were performed in PBS buffer (pH 7.0, 0.1 M) containing 0.1 M TEOA with 0.0 V applied voltage and 410 nm excitation light.

To evaluate the feasibility of the sensing platform, their PEC behaviors were then characterized by chronoamperometric *i*–*t* curves from the stepwise transient photocurrent responses upon intermittent light irradiation. As shown in Fig. 4a, the photocurrent of bare FTO is negligible (curve a), while g-C_3_N_4_/TiO_2_ NAs electrode exhibited the significantly strong photocurrent (curve b). Compared with g-C_3_N_4_/TiO_2_/FTO, the photocurrent response of Cu^2+^-doped g-C_3_N_4_/TiO_2_/FTO appeared a small decrease (curve c) which resulted from the capture of photoelectrons by Cu^2+^. And as can be seen from curve d, the photocurrent signal of Cu^2+^-doped g-C_3_N_4_/TiO_2_/FTO with the exposure of the electrode to a 50 nM Na_2_S solution caused a noticeable quenching, because of the generation of Cu_*x*_S which offered the trapping sites with new energy levels and thus suppressed the electron transfer on the surface of the g-C_3_N_4_/TiO_2_/FTO, as illustrated in Fig. 4b.^40^ In addition, the corresponding UV-vis diffuse reflectance spectra were also performed to further confirmed the enhanced absorption after the successful preparation of Cu^2+^-doped g-C_3_N_4_/TiO_2_/FTO, as shown in Fig. 4c. Fig. 4d indicated the signal response of g-C_3_N_4_/TiO_2_/FTO upon irradiation repeated every 10 s. The irradiation process was repeated over 400 s and no obvious variation could be observed, featuring the high stability of the biosensor. Thereby, all of these results demonstrated the feasible fabrication of the PEC sensing platform.

### 3.4 Analytical performance

**Fig. 5:**
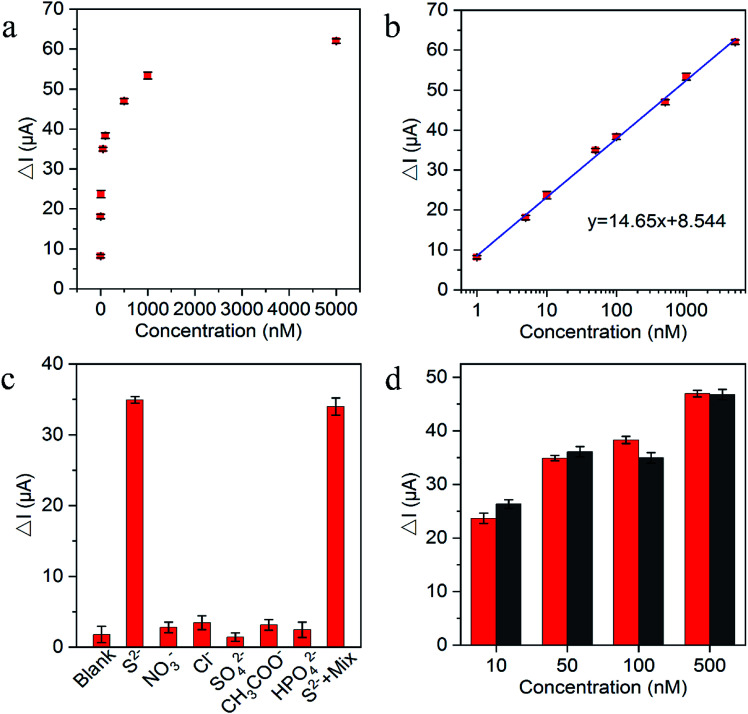
(a) Photocurrent intensities after incubation with increased S^2−^ concentration; (b) derived calibration curve; (c) selectivity of the proposed biosensor to H_2_S with 50 nM by comparing to the interference at 5 μM level: NO_3_^−^, Cl^−^, SO_4_^2−^, CH_3_COO^−^ and HPO_4_^2−^; (d) photocurrent decrement corresponding to different H_2_S concentrations in PBS (black) and in normal human plasma (red). Δ*I* is the photocurrent decrement corresponding to the various S^2−^ concentrations.

The substantial photocurrent decrement in the presence of trace amounts of the H_2_S captured in an aqueous medium (for convenience, Na_2_S was used as the source here) demonstrated the suitability of the fabricated biosensor for S^2−^ and H_2_S determination. Fig. 5a exhibited the decrement of photocurrent after reaction with various S^2−^ concentrations. Fig. 5b shows the photocurrent decrement linearly increased with the increasing S^2−^ concentrations from 1 × 10^−9^ to 5 × 10^−6^ M and the lowest detection limit of S^2−^ was estimated at 5.8 × 10^−11^ M (S/N = 3), which was comparable to other H_2_S PEC sensors in Table 1. As demonstrated in Table 1, different than the common zero-dimensional and one-dimensional material-based heterojunction photoelectrode, g-C_3_N_4_/TiO_2_/FTO as the representative two-dimensional material-based heterojunction photoelectrode truly has the relatively better photoelectric conversion efficiency and perfectly fits for H_2_S detection with *in situ* quenching strategy. The reproducibility of the PEC biosensor was assessed on the basis of the relative standard deviation (RSD) for the intra-assay and interassay precision. The intra-assay precision was obtained by parallel measuring S^2−^ five times at concentrations of 10 nM, 50 nM, and 100 nM, which yielded a RSD values of 4.0%, 3.6%, and 5.2%, respectively. The interassay precision was determined by assaying S^2−^ at the same concentration using five sensing electrodes prepared under identical conditions, where the RSD values were 5.8%, 4.6%, and 5.4%, respectively. These results indicated the satisfactory precision and reproducibility of this biosensor. To verify the selectivity of the PEC sensor, the common anions and other species potentially coexisting in the solution, including NO_3_^−^, Cl^−^, SO_4_^2−^, CH_3_COO^−^ and HPO_4_^2−^ were selected for interference test. As displayed in Fig. 5c, the photocurrent response to the interfering ions with the addition of 100-fold excess in comparison with S^2−^ were very close to the blank test, because of the interfering ions cannot have the reaction with Cu^2+^ to *in situ* generate the substrate insoluble in aqueous solution. Moreover, the photocurrent of the mixture containing S^2−^ was approximately the same as pure S^2−^, indicating that the coexistence of the S^2−^ with the interfering ions did not have a significant effect on the photocurrent of the sensing platform. Additionally, the long term stability performance of the designed sensing platform was also evaluated. There was no apparent change of the photocurrent response after the biosensor was stored at 4 °C in a refrigerator for over 1 month, and 94.4% of the initial photocurrent response was maintained after storage for over 2 months, suggesting the robustness of the as-designed PEC sensor. For the feasibility of practical application, the performance of the sensing platform was tested in human plasma. Different concentrations of mixed anions were then added. As shown in Fig. 5d, the small signal difference between normal human plasma and Tris–HCl solution samples indicated the precision of the sensing platform and the potential for practical applications.

**Table 1:** Comparison of some recent H_2_S PEC sensors

PEC substrate	Liner range (nmol mL^−1^)	LOD (nmol mL^−1^)	References
Cu^2+^ doped g-C_3_N_4_/TiO_2_ NAs	1–5000	0.058	This work
N–C dots/TiO_2_ NWs	10–100 000	10	20
Cd^2+^/TiO_2_ NTs	10–1 000 000	0.31	21
Cd^2+^/branched TiO_2_ NRs	1–5 000 000	29 ng mL^−1^	22
Cd^2+^/TiO_2_ NTs	10–1 000 000	10	23
Cd^2+^/TiO_2_ NTs	10–1 000 000	0.7	24

## 4. Conclusions

In summary, we successfully designed and fabricated a novel and general PEC sensing platform for highly sensitive H_2_S detection based on Cu^2+^-doped g-C_3_N_4_/TiO_2_ NAs heterojunction photoelectrode, *in situ* formed Cu_*x*_S would open a new pathway for the electron−hole recombination and thus efficiently inhibit the photocurrent generation of the sensing platform. Importantly, the above biosensor was highly sensitive and easy to prepare, manifesting a wide linear response range with S^2−^ detection limit of 58 pM. This study displayed the desirable potential of g-C_3_N_4_/TiO_2_ as the representative 2D material-based heterojunction in improving performance of PEC detection and was expected to inspire more interests in the implementation of numerous other semiconductor applications. Further work will focus on experimental optimization for better performance.

## Conflicts of interest

There are no conflicts to declare.

## Supplementary Material
